# Mechanism of Ca^2+^ transport by ferroportin

**DOI:** 10.7554/eLife.82947

**Published:** 2023-01-17

**Authors:** Jiemin Shen, Azaan Saalim Wilbon, Ming Zhou, Yaping Pan

**Affiliations:** 1 Verna and Marrs McLean Department of Biochemistry and Molecular Biology, Baylor College of Medicine Houston United States; Stockholm University Sweden; National Institute of Neurological Disorders and Stroke, National Institutes of Health United States

**Keywords:** SLC40, structure, cryo-EM, membrane transport, calcium, iron, Human

## Abstract

Ferroportin (Fpn) is a transporter that releases ferrous ion (Fe^2+^) from cells and is important for homeostasis of iron in circulation. Export of one Fe^2+^ by Fpn is coupled to import of two H^+^ to maintain charge balance. Here, we show that human Fpn (HsFpn) binds to and mediates Ca^2+^ transport. We determine the structure of Ca^2+^-bound HsFpn and identify a single Ca^2+^ binding site distinct from the Fe^2+^ binding sites. Further studies validate the Ca^2+^ binding site and show that Ca^2+^ transport is not coupled to transport of another ion. In addition, Ca^2+^ transport is significantly inhibited in the presence of Fe^2+^ but not vice versa. Function of Fpn as a Ca^2+^ uniporter may allow regulation of iron homeostasis by Ca^2+^.

## Introduction

Ferroportin (Fpn), encoded by the *SLC40A1* gene, is the only known Fe^2+^ exporter in human (Nemeth and Ganz, 2021; Abboud and Haile, 2000; Donovan et al., 2000; McKie et al., 2000). Fpn is highly expressed on enterocytes, hepatocytes, and macrophages, and mediates release of iron stored in cells (Drakesmith et al., 2015; Mackenzie and Garrick, 2005; Knutson et al., 2005). Activity of Fpn is regulated by hepcidin, a small peptide hormone secreted by hepatocytes, which binds to Fpn and reduces iron export by inhibiting its transport activity and triggering endocytosis of Fpn (Aschemeyer et al., 2018; Nemeth et al., 2004; Nemeth and Ganz, 2021). Mutations in Fpn cause hereditary hemochromatosis and iron-loading anemias in human (Pietrangelo, 2017; Ganz, 2013; Ginzburg, 2019; Vlasveld et al., 2019).

We have shown previously that Fpn is a Fe^2+^/2H^+^ exchanger, that is, the export of one Fe^2+^ is accompanied by the import of two H^+^ (Pan et al., 2020). The electroneutral transport mechanism is likely an adaptation to overcome the negative resting membrane potential that would have significantly hindered export of cations. Structures of mammalian Fpn show two transition-metal ion binding sites, termed Site 1 (S1) and Site 2 (S2) (Billesbølle et al., 2020; Pan et al., 2020). Curiously, each ion binding site is composed of only two residues, Asp39 and His43 for S1, and Cys326 and His507 for S2, and it remains unsettled how S1 and S2 mediate coupled transport of Fe^2+^ and H^+^. In the current study, we found that Asp39 is also part of a Ca^2+^ binding site, and that mutation Asp39Ala almost completely eliminates Ca^2+^ transport but has a modest impact on Fe^2+^ transport, suggesting that S1 and S2 may have different roles in Fe^2+^ or H^+^ transport.

Ca^2+^ is known to bind to mammalian Fpn (Deshpande et al., 2018), but Ca^2+^ transport by Fpn has not been demonstrated and the role of Ca^2+^ in Fe^2+^ transport remains ambiguous. Using Fpn expressed in *Xenopus* oocytes, Deshpande et al. found that Fe^2+^ export requires Ca^2+^, but Ca^2+^ is not transported by Fpn (Deshpande et al., 2018). More recently, several studies using purified Fpn reconstituted into liposomes found that Fe^2+^ transport occurs in the absence of Ca^2+^ (Li et al., 2020; Pan et al., 2020; Billesbølle et al., 2020), and that Ca^2+^ could potentiate Fe^2+^ transport under certain conditions (Li et al., 2020; Billesbølle et al., 2020). In the current study, we visualize the Ca^2+^ binding site in HsFpn and we demonstrate that Ca^2+^ is transported by Fpn. We then examine the mechanism of Ca^2+^ transport and its effect on Fe^2+^ transport.

## Results

### HsFpn is a Ca^2+^ uniporter

**Figure 1.**
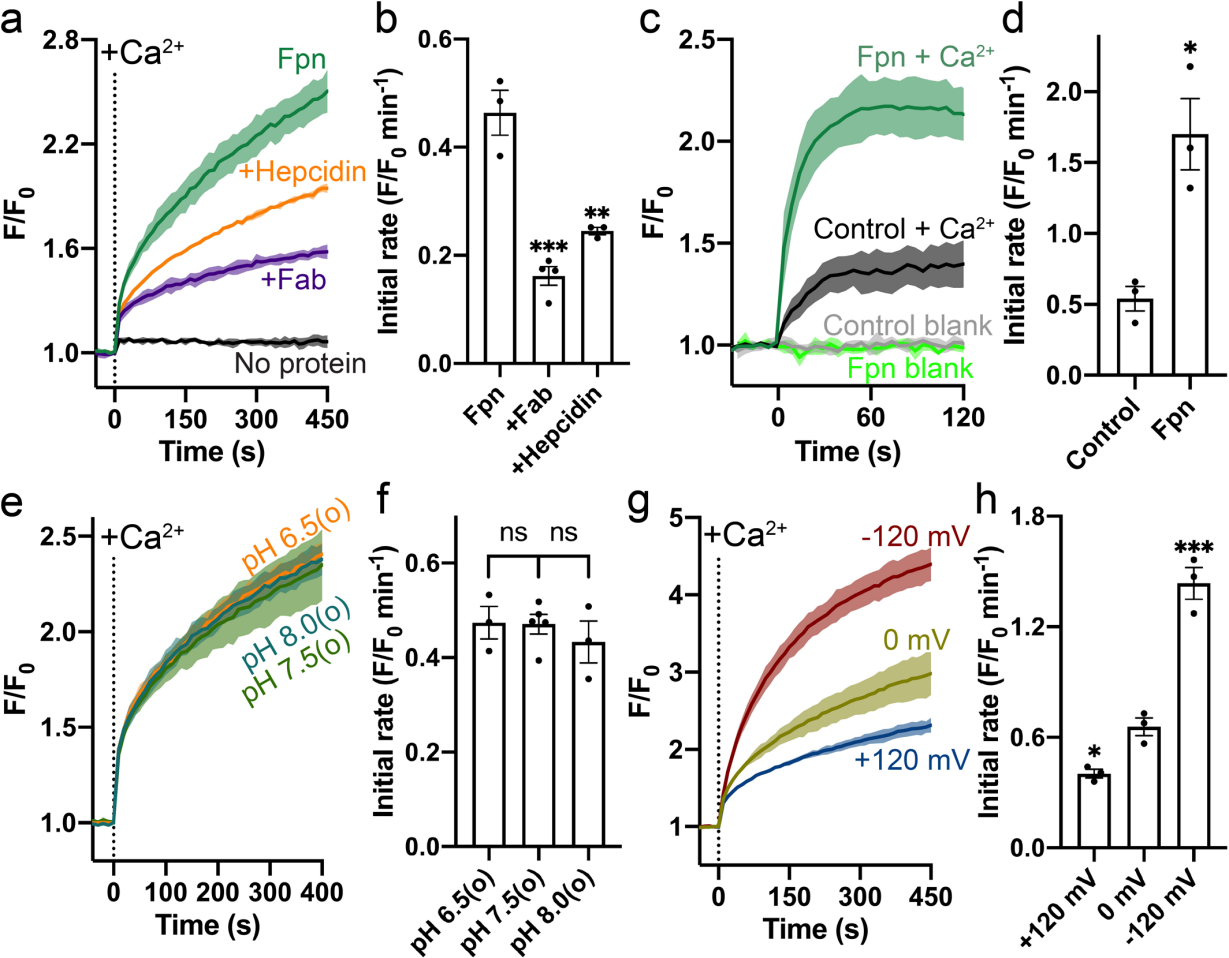
Specific Ca^2+^ uniport by HsFpn. (**a**) Ca^2+^ influx by HsFpn in proteoliposomes measured by fluorescence changes (F/F_0_) of Fluo-4 (green). The presence of 11F9 Fab (purple) or hepcidin (orange) reduces the Ca^2+^ influx. (**b**) Comparison of initial rates of Ca^2+^ influx in (**a**). (**c**) Ca^2+^ uptake in HEK cells expressing HsFpn monitored by Fluo-4 loaded inside cells. Cells overexpressing Fpn show a significantly faster Ca^2+^ uptake compared to control cells transfected with an empty vector. (**d**) Comparison of initial rates of Ca^2+^ uptake in (**c**). (**e**) Ca^2+^ influx by HsFpn in proteoliposomes at different outside (**o**) pHs. The inside pH is maintained at 7.5. (**f**) Comparison of initial rates of Ca^2+^ transport at different outside pHs. (**g**) Ca^2+^ influx by HsFpn in proteoliposomes at different membrane potentials. Valinomycin was used to generate a membrane potential prior to the addition of Ca^2+^. (**h**) Comparison of initial rates of Ca^2+^ transport at different membrane potentials. In all the transport assays, 500 µM of Ca^2+^ was added at time zero. Statistical significances were analyzed with one-way analysis of variance (ANOVA) followed by Dunnett’s test for multiple comparisons. In this article, all time-dependent fluorescence traces are shown as solid lines (mean) with shaded regions (standard deviation, SD) from at least three biological repeats. For all bar graphs, a scatter plot is overlaid on each bar. The height represents the mean of at least three measurements, and the error bar standard error of the mean (SEM). Statistical significances are indicated as follows: ns, not significant; *, p < 0.05; **, p < 0.01; ***, p < 0.001; ****, p<0.0001.

**Figure 2.**
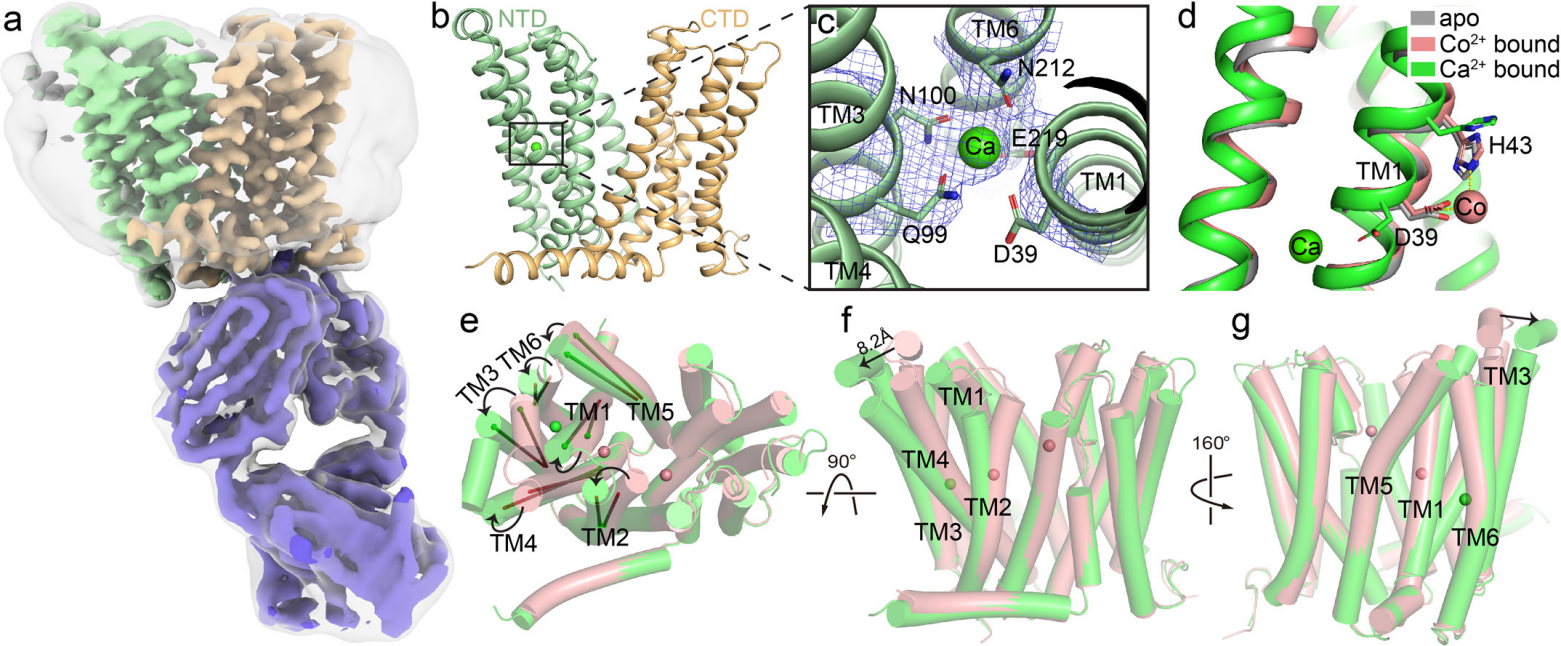
Structure of HsFpn bound to Ca^2+^. (**a**) Cryo-EM map of HsFpn in complex with 11F9 in the presence of Ca^2+^. Densities for NTD, CTD, and 11F9 are colored in pale green, light orange, and slate purple, respectively. A smoothened map contoured at a low threshold (translucent grey) is overlaid to show the lipid nanodisc density around Fpn. (**b**) Structure of HsFpn with a bound Ca^2+^ in an outward-facing conformation. NTD and CTD are colored as described in (**a**). Ca^2+^ is shown as a green sphere. (**c**) Zoomed-in view of the Ca^2+^ binding site in the NTD. The five residues coordinating Ca^2+^ are labeled and shown as side chain sticks. The density for Ca^2+^ is contoured at 7.5σ as blue mesh. (**d**) Structural comparison of apo (grey, PDB ID 6W4S), Co^2+^-bound (pink, PDB ID 8DL8), and Ca^2+^-bound (green) HsFpn near S1. The side chains of D39 and H43 are shown as sticks. Co^2+^ is shown as a pink sphere. (**e**), (**f**), and (**g**) Three views of conformational changes in NTD induced by Ca^2+^ binding. The Co^2+^-bound (pink) and Ca^2+^-bound (green) structures are aligned and shown as cartoon with cylindrical helices. (**e**) Top view (from the extracellular side) of the alignment. The helical directions of TM1-6 are visualized by vectors inside cylinders, and the bending of these helices is indicated by black arrows. Bending of TMs viewed from the front (**f**) and the back (**g**). The displacement of the extracellular loop between TM3 and TM4 is marked with a black arrow and distance.

HsFpn was expressed, purified, and reconstituted into liposomes for transport assays (Figure 1—figure supplement 1a and Materials and methods). Significant Ca^2+^ uptake was observed in proteoliposomes reconstituted with HsFpn, as indicated by increased fluorescence of Fluo-4 trapped inside of the vesicles (Figure 1a and Figure 1—figure supplement 1b). In contrast, control liposomes that do not have HsFpn show minimal change in fluorescence (Figure 1a). We also found that Ca^2+^ transport is inhibited by hepcidin and by a monoclonal antibody (11F9) that binds to HsFpn with nanomolar affinity (Figure 1a–b), providing further support that HsFpn mediates Ca^2+^ transport. As the fragment of antigen-binding (Fab) of 11F9 is known to target the intracellular side of HsFpn (Figure 2a; Pan et al., 2020; Wilbon et al., 2023), we applied the Fab either to only the external side of the liposomes or to both sides of the liposomes, and found that the inhibition is significantly higher when Fab is present on both sides. This result indicates that HsFpn is incorporated into liposomes in both orientations (Figure 1—figure supplement 1c–g). We also examined HsFpn expressed in human embryonic kidney (HEK) cells and found that HsFpn mediates Ca^2+^ uptake (Figure 1c–d).

**Figure 1—figure supplement 1.**
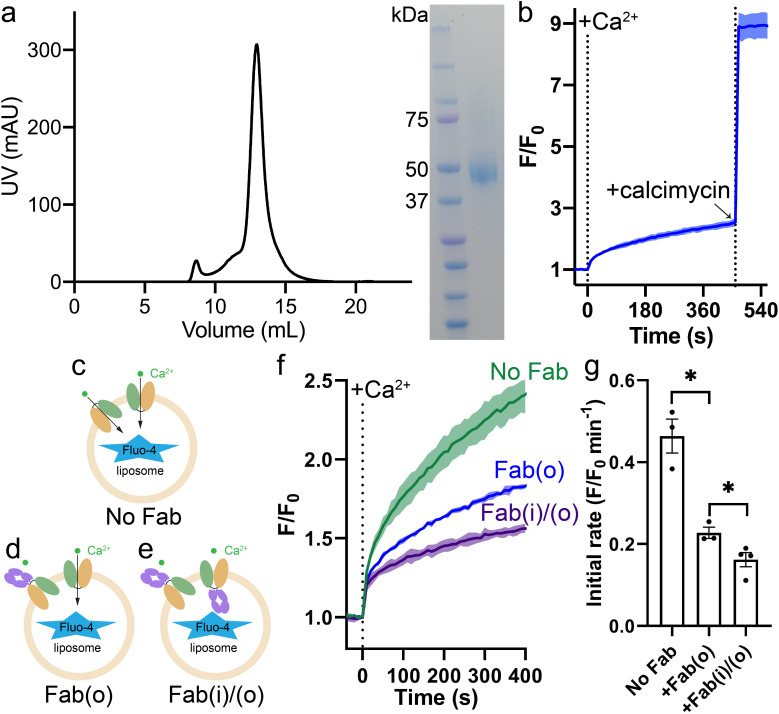
Expression of HsFpn and validation of Ca^2+^ transport in proteoliposomes. (**a**) SEC profile (left) and SDS-PAGE gel image (right) of purified HsFpn. (**b**) Calcimycin, a Ca^2+^ ionophore, collapses the Ca^2+^ gradient after 450 s of Ca^2+^ influx by Fpn in proteoliposomes. Schematics of Ca^2+^ transport by Fpn (pale green and light orange lobes) in proteoliposomes with (**c**) no Fab; (**d**) Fab (slate purple lobes) on the outside (**o**); (**e**) Fab on both the inside and outside (i/o). (**f**) Inhibition of Fpn-mediated Ca^2+^ influx in proteoliposomes by Fab added to different sides. (**g**) Comparison of initial rates of Ca^2+^ influx in (**f**). Unpaired t-test with Welch correction was used to analyze statistical significances. In (**b**) and (**f**), 500 µM of Ca^2+^ was added at time zero. Figure 1—figure supplement 1—source data 1.Uncropped SDS-PAGE gel image of purified HsFpn.Black dashed rectangle indicates the region of interest.

**Figure 1—figure supplement 2.**
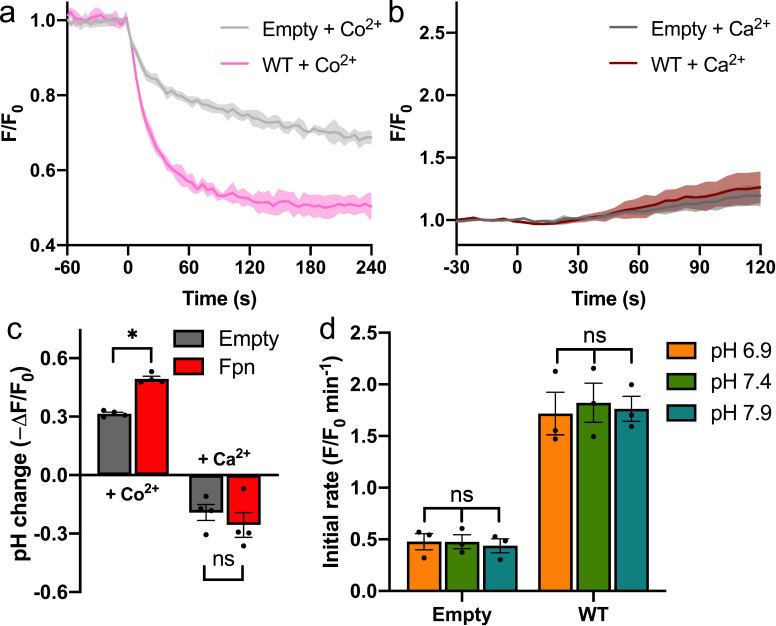
H^+^-independent Ca^2+^ uptake by HsFpn in HEK cells. Intracellular pH change induced by addition of Co^2+^ (**a**) or Ca^2+^ (**b**) on the extracellular side monitored by a pH-sensitive dye (pHrodo Red). (**c**) Comparison of cytosolic pH changes induced by the Co^2+^ or Ca^2+^ uptake. The mean final fluorescence changes from the traces in (**a**) and (**b**) are plotted. Decreased relative fluorescence (ΔF/F_0_) correlates to increased pH. Sidak’s multiple comparisons test was used following two-way ANOVA. (**d**) Ca^2+^ transport rates at different extracellular pHs. Two-way ANOVA: among different pHs, p=0.912; between empty and WT, p<0.0001; interaction, p=0.918.

**Figure 1—figure supplement 3.**
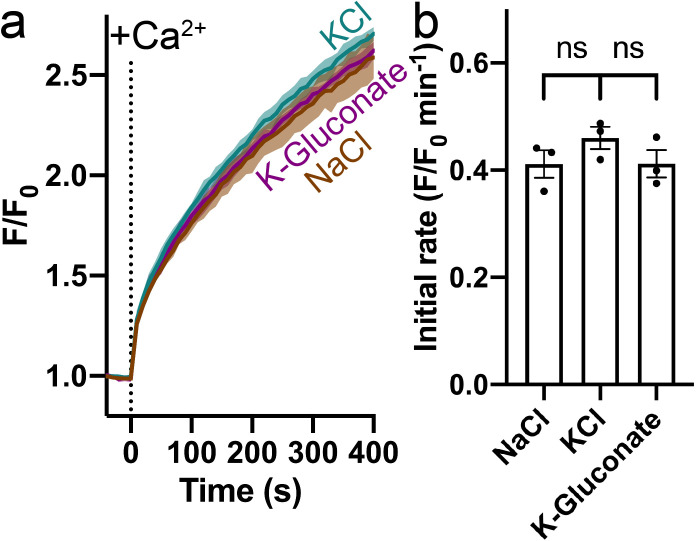
Effect of Na^+^, K^+^, and Cl^-^ on Ca^2+^ transport by HsFpn. (**a**) Ca^2+^ influx in proteoliposomes with symmetrical NaCl, KCl, and K-Gluconate. 500 µM Ca^2+^ was added at time zero. (**b**) Comparison of initial rates of Ca^2+^ transport in different salts. Statistical significances were analyzed with one-way ANOVA.

**Figure 1—figure supplement 4.**
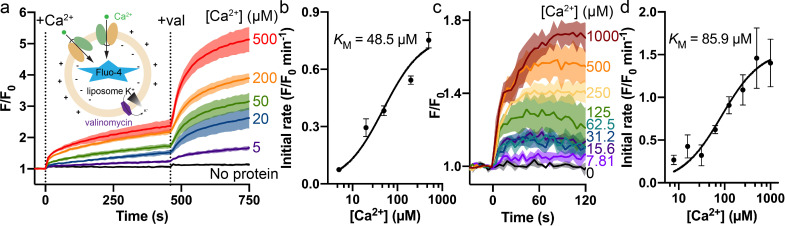
Dose-dependent Ca^2+^ transport by Fpn. (**a**) Ca^2+^ transport into liposome with purified Fpn monitored by impermeable Fluo-4. Different concentrations of Ca^2+^ (labeled to the right of each trace) were added followed by the addition of valinomycin (val) to clamp membrane potential. The time points for the addition of Ca^2+^ or valinomycin are indicated by vertical dashed lines. For the ‘No protein’ control, 500 µM Ca^2+^ was used. The inset shows the schematic of the proteoliposomes with a 100:1 inside:outside K^+^ gradient. (**b**) Initial rates of fluorescence change after the addition of valinomycin versus concentrations of Ca^2+^ for data in (**a**). (**c**) Dose-dependent Fpn-specific Ca^2+^ uptake in HEK cells expressing Fpn measured by Fluo-4 loaded inside cells. The raw traces of the empty control group at a certain [Ca^2+^] were subtracted from the corresponding traces of the Fpn WT group. (**d**) Initial rates of fluorescence change versus concentrations of Ca^2+^ in HEK cells. The black solid lines in (**b**) and (**d**) are curve fits to a Michaelis–Menten equation.

**Figure 2—figure supplement 1.**
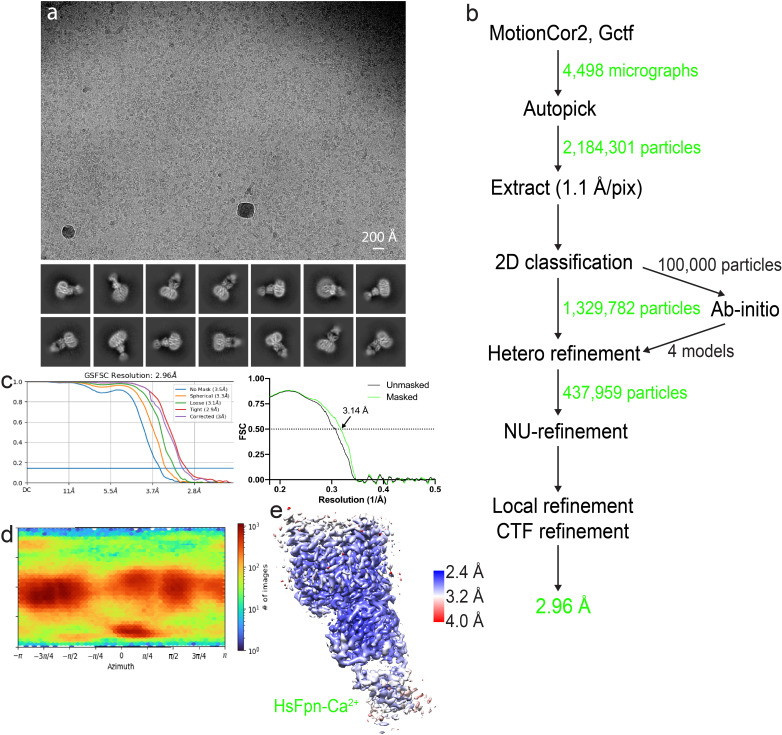
Cryo-EM analysis of HsFpn-Ca^2+^ in nanodisc. (**a**) Representative electron micrograph (upper panel) and 2D class averages (lower panel). (**b**) Workflow of single-particle data processing. (**c**) The gold-standard Fourier shell correlation (FSC) curves (left panel) and map-to-model FSC curves (right panel). (**d**) Direction distribution of particles used in the final 3D reconstruction. (**e**) Local resolution map.

**Figure 2—figure supplement 2.**
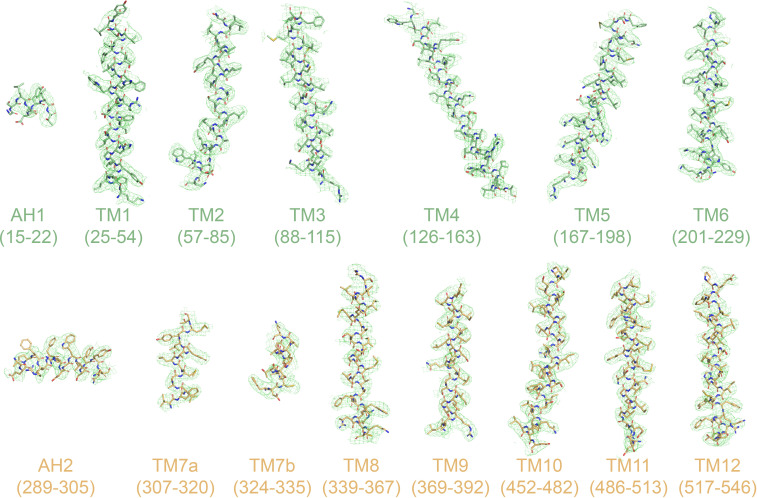
Cryo-EM densities of TM helices and amphipathic helices (AH). Densities for HsFpn-Ca^2+^ are shown as green mesh. Residues within the ranges indicated below are rendered in stick representation and colored in pale green for NTD or light orange for CTD.

**Figure 2—figure supplement 3.**
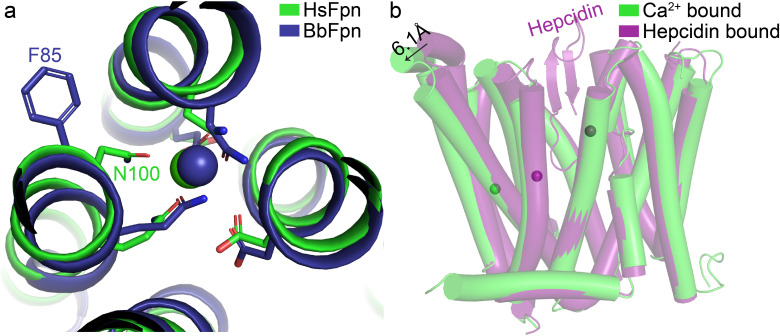
Structural comparisons of Ca^2+^-bound HsFpn. (**a**) Differences in Ca^2+^ coordination by HsFpn (green) and by BbFpn (deep blue, PDB ID 6BTX). (**b**) Comparison of Ca^2+^-bound (green) versus hepcidin-bound (deep purple, PDB ID 6WBV) structure of HsFpn shows further outward movements of NTD. Displacement of the loop between TM3 and TM4 is marked with an arrow and distance. Co^2+^ ions in the Fpn-hepcidin structure are shown as deep purple spheres.

To understand the mechanism of Ca^2+^ transport by HsFpn, we first examined whether common ions, H^+^, Na^+^, K^+^, and Cl^-^, are involved or required for Ca^2+^ transport. We found that Ca^2+^ transport by HsFpn is not affected by a H^+^ gradient (Figure 1e–f and Figure 1—figure supplement 2), and that the absence of Na^+^, K^+^, or Cl^-^ has no significant effect on Ca^2+^ transport (Figure 1—figure supplement 3). These results suggest that Fpn is a Ca^2+^ uniporter. If this is true, then Ca^2+^ transport should be electrogenic and sensitive to membrane potentials. We tested this in the following experiments. We measured Ca^2+^ transport at defined membrane potentials,+120 mV, 0 mV, and –120 mV, established by a K^+^ gradient in the presence of valinomycin, a K^+^ selective ionophore (Figure 1g). As shown in Figure 1g–h, Ca^2+^ transport is significantly enhanced at –120 mV and reduced at +120 mV, and Ca^2+^ transport at 0 mV is larger than that in the absence of a clamped membrane potential. These results are fully consistent with HsFpn being a Ca^2+^ uniporter.

We then measured Ca^2+^ transport at different concentrations of Ca^2+^ ([Ca^2+^]), first in the absence of a preset membrane potential and then in the presence of –120 mV membrane potential (Figure 1—figure supplement 4a). We first monitored Ca^2+^ influx in the presence of a 100-fold K^+^ concentration gradient but in the absence of valinomycin. Under this condition, import of Ca^2+^ would build up a positive membrane potential that slows down further Ca^2+^ influx. After the influx reached a steady state (~460 s), we added valinomycin to clamp the membrane potential to –120 mV and we observed a large increase of Ca^2+^ influx. The rate of Ca^2+^ uptake in the second phase is faster than that in the first phase (Figure 1—figure supplement 4a). These results are consistent with HsFpn being a Ca^2+^ uniporter. The Michaelis-Menten constant, *K*_M_, for Ca^2+^ transport, calculated based on the initial rate of fluorescence increase in the second phase, is 48.5 (27.3–88.8) µM (95% confidence interval in parentheses) (Figure 1—figure supplement 4b). We also measured Ca^2+^ uptake in HEK cells expressing HsFpn and obtained a *K*_M_ of 85.9 (36.6–193.0) µM (Figure 1—figure supplement 4c–d).

### Structure of HsFpn bound to Ca^2+^

We determined the structure of nanodisc-enclosed HsFpn-11F9 complex in the presence of 2 mM Ca^2+^ by cryo-electron microscopy (cryo-EM) to an overall resolution of 3.0 Å (Figure 2a and Figure 2—figure supplement 1, Table 1). The density map reaches a resolution of ~2.4 Å in the transmembrane (TM) regions and allows for the building and refinement of a structural model that contains residues 15–240, 289–400, and 452–555, which covers all 12 TM helices (Figure 2—figure supplement 2). Residues 1–14, 241–288, 401–451, and 556–571, which are predicted to be disordered regions located to either the N- or C-terminus or loops between TM helices, are not resolved. The 12 TMs form two well-defined domains, with the N-terminal domain (NTD) composed of TM1–6 and the C-terminal domain (CTD) TM7–12 (Figure 2b). The current structure assumes an outward-facing conformation in which the NTD and CTD make contact on the intracellular side. The overall conformation of the Ca^2+^-bound HsFpn is similar to that of the Co^2+^-bound HsFpn (Wilbon et al., 2023; Figure 2e–g and Figure 2—figure supplement 3b).

**Table 1.** Summary of cryo-EM data collection, processing, and refinement.

Sample	HsFpn-Ca^2+^–11 F9
**Cryo-EM data collection**	
Voltage (kV)	300
Magnification (×)	81,000
Pixel Size (Å)	1.10
Electron exposure (e^-^/Å^2^/frame)	1.25
Defocus range (µm)	[-2.5,–0.8]
Number of image stacks	4,498
Number of frames per stack	40
**Cryo-EM data processing**	
Initial number of particles	2,184,301
Final number of particles	437,959
Symmetry imposed	C1
Map resolution (Å)	3.0
Map resolution range (Å)	2.4–3.6
FSC threshold	0.143
**Model refinement**	
Number of amino acids	875
Total non-hydrogen atoms	6,192
Average B factor (Å^2^)	171.4
Bond length RMSD (Å)	0.008
Bond angle RMSD (°)	0.939
Ramachandran Plot	
Favored (%)	93.53
Allowed (%)	6.24
Outliers (%)	0.23
Rotamer outliers (%)	1.25
MolProbity Score	1.98

In the density map (Figure 2a), we noticed a non-protein density corralled by four helices (TM1, TM3, TM4, and TM6) in the NTD, and assigned it as a Ca^2+^ based on the following observations. This density was not present in previous structures of Co^2+^-bound HsFpn or a highly homologous Fpn from Tarsier monkey (Pan et al., 2020; Billesbølle et al., 2020; Wilbon et al., 2023). The side chains of five conserved residues, Asp39, Gln99, Asn100, Asn212, and Glu219, are within 4 Å of the presumed Ca^2+^ density (Figure 2b–c). The side chain positions of Gln99, Asn100, and Asn212 are defined by the density map, while these of Asp39 and Glu219 are deduced based on their backbone and partially resolved side chain densities. While in structures of Co^2+^-bound HsFpn (Wilbon et al., 2023; Billesbølle et al., 2020), the side chains of Asp39 and His43 point towards the central cavity between the NTD and CTD to form S1, the side chain of Asp39 in the current structure points towards the interior of the four-helical bundle to coordinate Ca^2+^ (Figure 2d). His43 also assumes a different rotamer conformation in the current structure (Figure 2d). When the NTD and CTD are aligned individually with their counterparts in the Co^2+^-bound HsFpn (Wilbon et al., 2023), the NTD has a root mean squared distance (RMSD) of 1.32 Å and the CTD 0.51 Å. The larger RMSD in the NTD is due to deviations of the extracellular halves of the TM helices and the extracellular loop between TM3–4 (Figure 2e–g). The bound Ca^2+^ is not solvent-accessible in the current structure.

### Validation of the Ca^2+^ binding site

**Figure 3.**
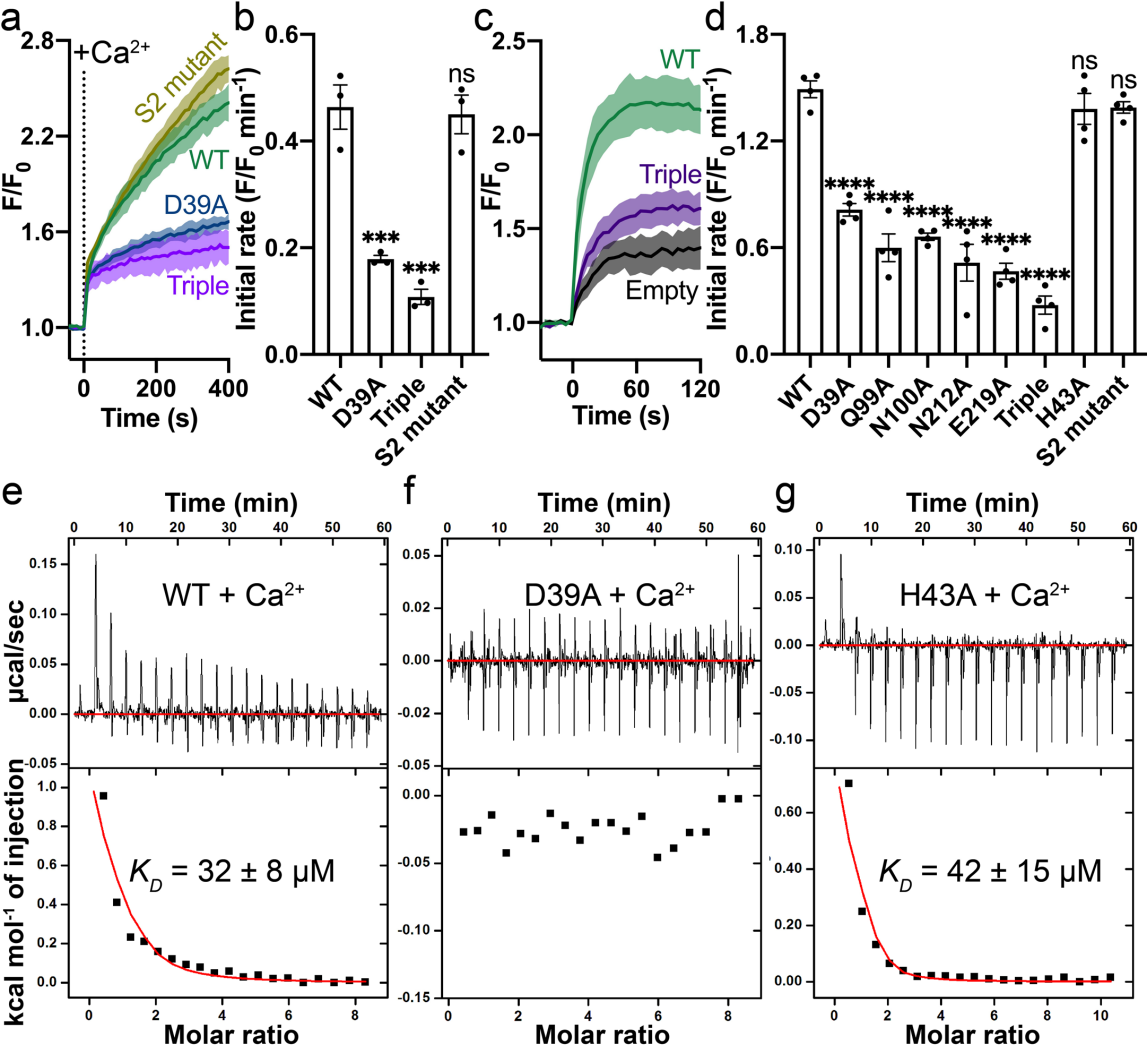
Mutations on the Ca^2+^ binding site. (**a**) Ca^2+^ influx by WT (green), D39A (blue), and the triple mutant (Q99A_N100A_E219A, purple) of the Ca^2+^ binding site in proteoliposomes. (**b**) Comparison of initial rates of Ca^2+^ influx in (**a**). (**c**) Ca^2+^ uptake in HEK cells expressing WT (green) or the triple mutant (purple) Fpn. (**d**) Fpn-specific Ca^2+^ transport activities of WT and mutants. Initial rates are subtracted from the empty control. For statistical significances in (**b**) and (**d**), Dunnett’s test was used as a post hoc test following one-way ANOVA with the WT as the control. 500 µM Ca^2+^ was used in (**a**–**d**). Binding of Ca^2+^ to WT (**e**), D39A (**f**), and H43A (**g**) HsFpn measured by ITC. Upper plot: raw thermogram showing the heat during binding and baseline (red line). Lower plot: integrated heat of each injection (black square) and the fit of data (red line).

We validated the Ca^2+^ binding site by mutational studies. Both a single alanine mutation (Asp39Ala) and a triple alanine mutation (Gln99_Asn100_Glu219 to Ala) to the Ca^2+^ binding site show significantly reduced Ca^2+^ transport activity in the proteoliposome assay (Figure 3a–b). In contrast, the double alanine mutation to S2 (Cys326Ala_His507Ala) does not affect Ca^2+^ transport significantly (Figure 3a–b). We also measured Ca^2+^ uptake by HsFpn mutants expressed in HEK cells (Figure 3—figure supplement 1) and found that single alanine mutations to residues at the Ca^2+^ binding site significantly impair Ca^2+^ transport activity, while mutation to His43 (at S1), which is one helical turn away from Asp39 at Ca^2+^ binding site, maintains high Ca^2+^ transport activity (Figure 3c–d).

**Figure 3—figure supplement 1.**
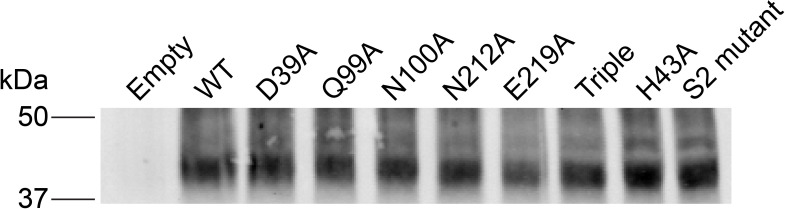
Expression of WT and mutant Fpns in HEK cells assessed by western blot. Figure 3—figure supplement 1—source data 1.Uncropped western blot image of Fpn expressed in HEK cells.Black dashed rectangle indicates the region of interest.

**Figure 3—figure supplement 2.**
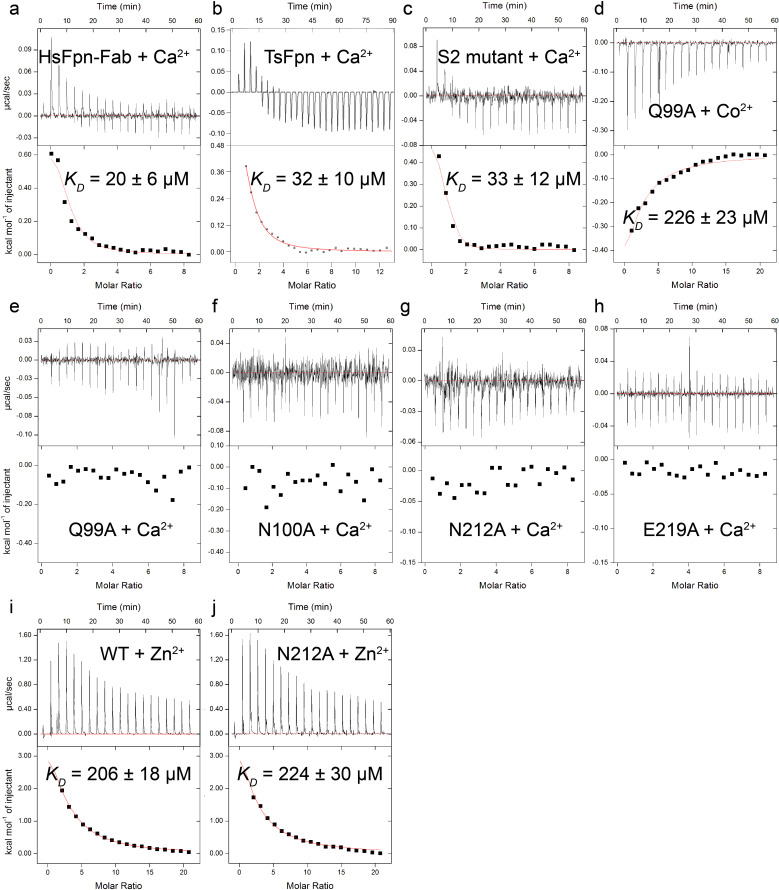
**Binding of Ca^2+^, Co^2+^**, **or Zn^2+^ to WT and mutant Fpns measured by ITC**. Binding of Ca^2+^ to HsFpn-11F9 complex (**a**), TsFpn (**b**), and S2 mutant (**c**). (**d**) Binding of Co^2+^ to the alanine mutant of Q99 in the Ca^2+^ binding site. Titration of Ca^2+^ into the alanine mutants of Q99 (**e**), N100 (**f**), N212 (**g**), and E219 (**h**) in the Ca^2+^ binding site. Binding of Zn^2+^ to WT (**i**) and the alanine mutant of N212 (**j**) in the Ca^2+^ binding site.

**Figure 3—figure supplement 3.**
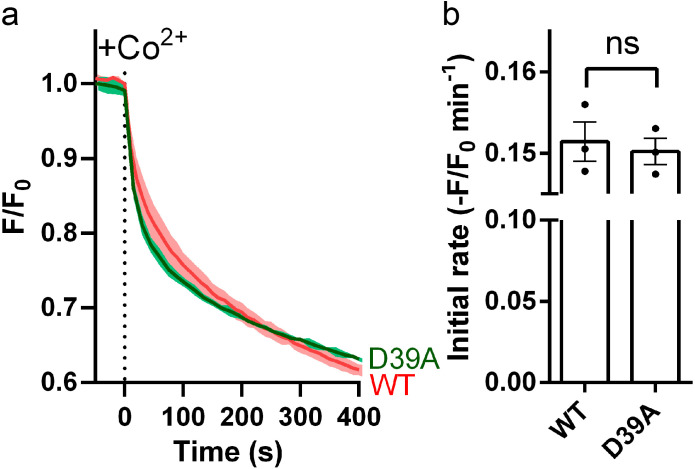
Effect of D39A on Co^2+^ transport by Fpn. (**a**) Co^2+^ influx in proteoliposomes with symmetrical NaCl buffer. 500 µM Co^2+^ was added at time zero. (**b**) Comparison of initial rates of Co^2+^ transport. Statistical significances were analyzed with unpaired t-test with Welch correction.

We estimated Ca^2+^ binding affinity by isothermal titration calorimetry (ITC). We found that Ca^2+^ binding is an endothermic process with a dissociation constant (*K_D_*) of ~32 µM (Figure 3e). Similar endothermic binding of Ca^2+^ was also reported in mouse Fpn and a bacterial homolog of Fpn (Deshpande et al., 2018). The 11F9 Fab does not interfere with or enhance Ca^2+^ binding as the HsFpn-11F9 complex has a similar Ca^2+^ binding affinity (*K_D_* = 20 ± 6 µM; ) (Figure 3—figure supplement 2a). We then measured Ca^2+^ binding to HsFpn mutants, and found that single point mutations to residues at the Ca^2+^ binding site abolish Ca^2+^ binding (Figure 3f and Figure 3—figure supplement 2e–h). As a control, we measured Ca^2+^ binding to the His43Ala mutant and to the S2 mutant, and found that both mutants retain Ca^2+^ binding with affinities similar to that of the wild-type (WT; Figure 3g and Figure 3—figure supplement 2c). In addition, a Ca^2+^ binding site mutant, Gln99Ala, does not significantly change Co^2+^ binding affinity (*K_D_* = 226 ± 23 µM; Figure 3—figure supplement 2d). Asn212Ala does not significantly change Zn^2+^ binding to HsFpn (*K_D_* = 224 ± 30 µM) either (Figure 3—figure supplement 2i–j). Combined, these results provide further validation to the observed Ca^2+^ binding site in HsFpn.

### Competition between Ca^2+^ and Co^2+^ in HsFpn

**Figure 4.**
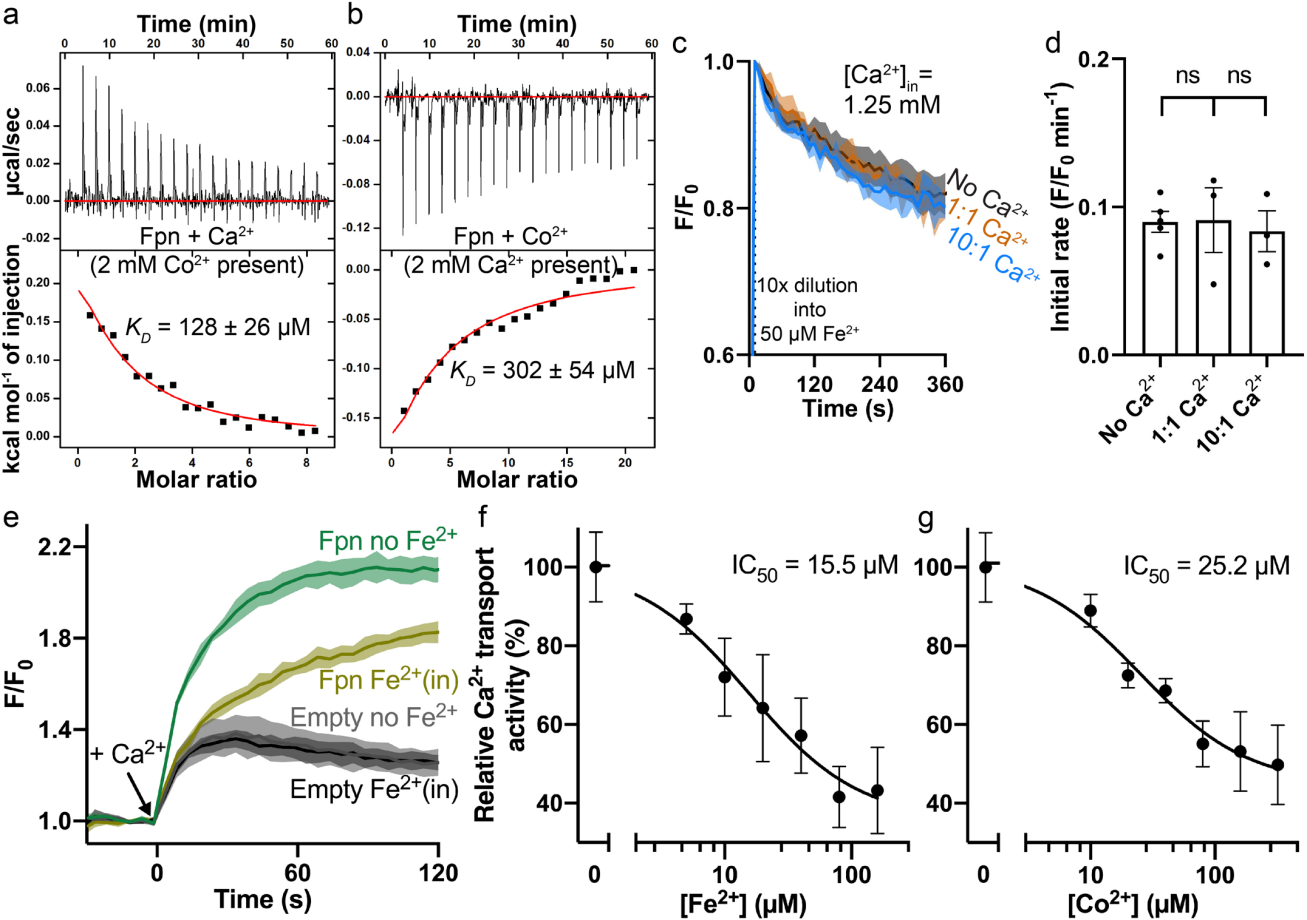
Interplay between Fe^2+^/Co^2+^ and Ca^2+^ in HsFpn. (**a**) Ca^2+^ binding in the presence of 2 mM Co^2+^. (**b**) Co^2+^ binding in the presence of 2 mM Ca^2+^. (**c**) Fe^2+^ transport into proteoliposomes in the presence or absence of Ca^2+^. The external [Fe^2+^] is 50 µM. ‘1:1’ indicates symmetrical [Ca^2+^] at 1.25 mM and “10:1” indicates 1.25 mM Ca^2+^ inside and 0.125 mM Ca^2+^ outside. 1 mM sodium ascorbate was included in all samples. All fluorescence traces are subtracted from corresponding blank controls using vesicles with no HsFpn. (**d**) Comparison of initial rates of Fe^2+^ transport in (**c**). One-way ANOVA was used for statistical analysis. (**e**) Ca^2+^ (1 mM) uptake by HEK cells expressing HsFpn in the presence (dark yellow) or absence (green) of 80 µM Fe^2+^ that has been pre-loaded into the cells. Uptake is monitored by jGCaMP7s which is co-expressed in cytosol. Cells transfected with an empty vector (dark and light gray) serve as negative controls. Ca^2+^ transport at different concentrations of Fe^2+^ (**f**) or Co^2+^ (**g**). Normalized initial rates of Fpn-specific Ca^2+^ uptake were used to represent relative Ca^2+^ transport activities. Data were fitted (black solid line) to an inhibitory dose-response equation to calculate IC_50_ values.

Next, we measured binding of Ca^2+^ in the presence of Co^2+^, and *vice versa*. In the presence of 2 mM Co^2+^, Ca^2+^ binding affinity is reduced by ~fourfold as shown in Figure 4a. The reduced binding is apparent both from the reduced heat absorption during the titration, and the reduced *K_D_* from fitting of the ITC data. We interpret the reduced Ca^2+^ binding in the presence of Co^2+^ as the loss of Asp39 to the coordination of Co^2+^ at S1 (Figure 2d). On the other hand, Co^2+^ binding affinity (*K_D_*) is not affected in the presence of 2 mM Ca^2+^, as shown in Figure 4b, although the amount of heat release is appreciably less in the presence of Ca^2+^. This result is similar to the effect of the S1 mutation on Co^2+^ binding (Asp39_His43 to Ala, *K_D_* = 266 ± 24 µM) (Pan et al., 2020). We interpret this result as the preservation of Co^2+^ binding at S2, which is not affected by Ca^2+^ binding.

**Figure 4—figure supplement 1.**
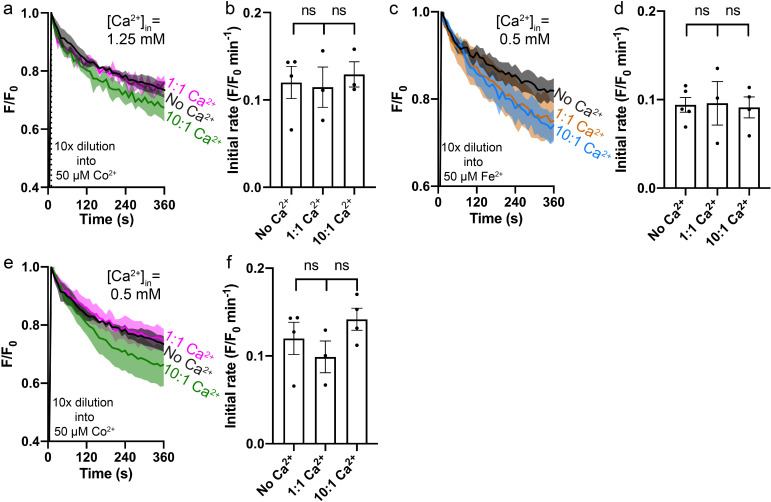
Fe^2+^/Co^2+^ transport into proteoliposomes by Fpn in the presence or absence of Ca^2+^. (**a**) Co^2+^ transport with or without 1.25 mM of intra-vesicular Ca^2+^. (**c**) Fe^2+^ transport with or without 0.5 mM of intra-vesicular Ca^2+^. (**e**) Co^2+^ transport with or without 0.5 mM of intra-vesicular Ca^2+^. (**b**), (**d**), and (**f**) Comparison of initial rates in (**a**), (**c**), and (**e**). Liposome samples were diluted (10×) into outside buffers containing 50 µM Co^2+^ or Fe^2+^. ‘1:1’ or ‘10:1’ denotes the ratio of [Ca^2+^] inside:outside achieved after 10×dilution. 1 mM sodium ascorbate was included in (**c**). All fluorescence traces were subtracted from their corresponding no protein controls. One-way ANOVA was used for statistical analysis.

**Figure 4—figure supplement 2.**
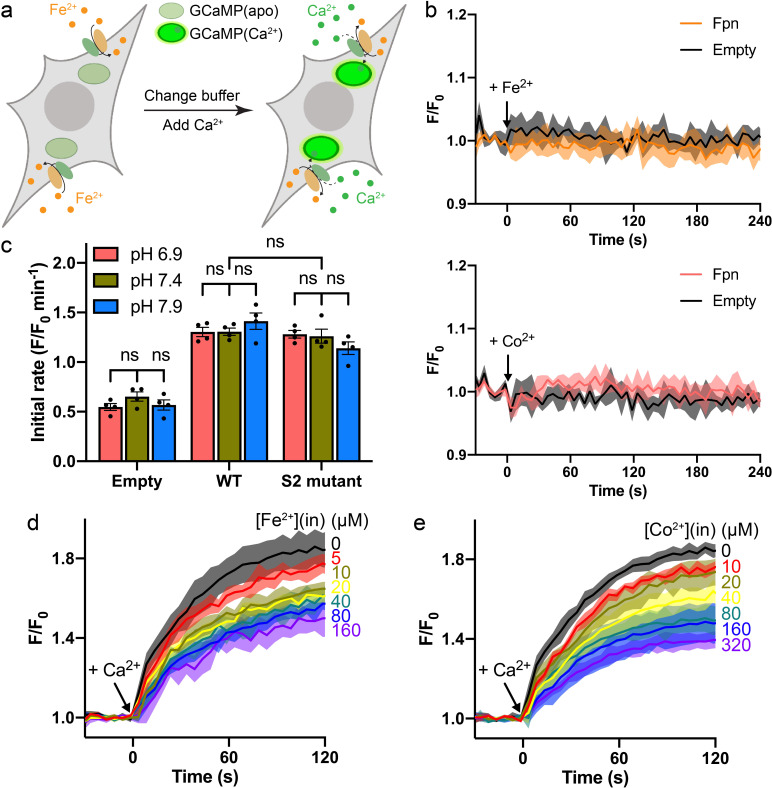
Ca^2+^ transport in the presence of Fe^2+^ or Co^2+^ in HEK cells. (**a**) Schematic of the Ca^2+^ transport assay in Fe^2+^-loaded HEK cells co-expressing Fpn and the Ca^2+^ sensor jGCaMP7s. (**b**) Transition metal ions do not interact with jGCaMP7s as indicated by unaltered fluorescence readings during loading of Fe^2+^ (top) or Co^2+^ (bottom) into HEK cells expressing Fpn (orange or pink traces) or transfected with empty vectors (gray traces). Black arrows indicate additions of metal ions. (**c**) Ca^2+^ transport at different extracellular pHs in the presence of 20 µM Co^2+^. Two-way ANOVA: among different pHs, p=0.717; among empty, WT, and S2 mutant, p<0.0001; interaction, p=0.130. (**d**) and (**e**) Fpn-specific Ca^2+^ transport in the presence of Fe^2+^ or Co^2+^ loaded inside HEK cells. The concentrations of Fe^2+^ or Co^2+^ used during the loading step are indicated to the right of each trace. The Fe^2+^ stock was prepared with a 10-fold molar excess of sodium ascorbate. The raw traces of the empty control group were subtracted from the corresponding traces of the Fpn WT group. Normalized initial rates calculated from the datasets are shown in Figure 4f–g.

**Figure 4—figure supplement 3.**
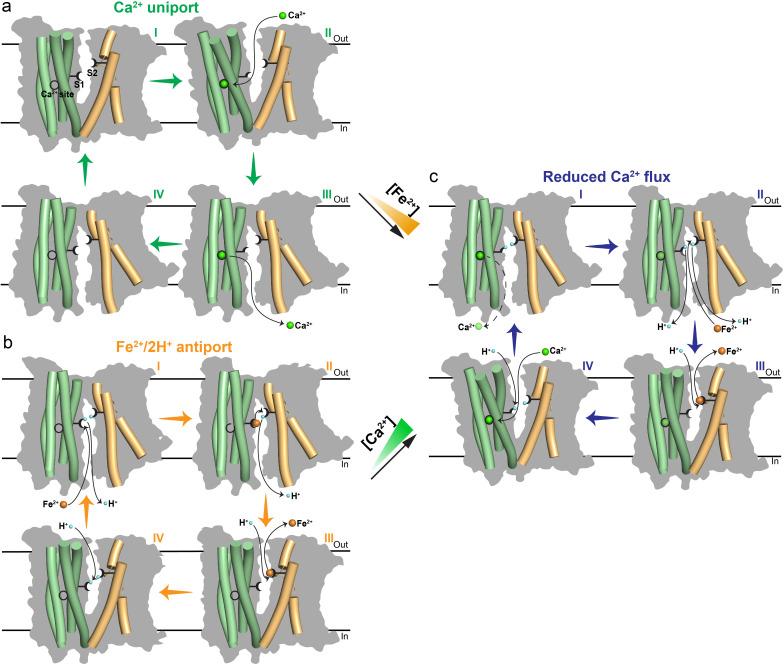
Proposed transport mechanisms of metal ions in Fpn. (**a**) Electrogenic Ca^2+^ uniport in the absence of Fe^2+^. (**b**) Electroneutral Fe^2+^/2H^+^ antiport in the absence of Ca^2+^. (**c**) Reduced Ca^2+^ flux in the presence of Fe^2+^. Higher concentration of Fe^2+^ (orange ramp) shows larger inhibition on Ca^2+^ transport. The inward-facing model is generated based on the bacterial homolog structure (PDB ID 6BTX). The four-helical bundle (pale green) in NTD, and TM7 and TM11 (light orange) in CTD are shown as cylinders and overlaid on silhouettes (grey) of the whole transporter. S1 and S2 of Fe^2+^ are indicated as black forks with two tines to represent two ligand residues in each site. The Ca^2+^ binding site is indicated as a black circle. When Fe^2+^ is bound to S1, the Ca^2+^ binding site is drawn as an incomplete circle to indicate its partial disruption. Similarly, the S1 becomes a half fork when Ca^2+^ is bound. H^+^ (blue), Fe^2+^ (orange), and Ca^2+^ (green) are shown as spheres. The black dashed line and shaded green spheres of Ca^2+^ in (**c**) indicate reduced Ca^2+^ release and occupancy.

We then measured Ca^2+^ transport in the presence of Fe^2+^ or Co^2+^, and Fe^2+^ or Co^2+^ transport in the presence of Ca^2+^. We found that Fe^2+^ or Co^2+^ transport is not affected significantly in the presence of either 1.25 mM or 0.5 mM Ca^2+^ (Figure 4c–d and Figure 4—figure supplement 1a–f), but Ca^2+^ transport is significantly reduced in the presence of Fe^2+^ or Co^2+^ (Figure 4e–g and Figure 4—figure supplement 2c–e). These results are consistent with the structures and our understanding of Fe^2+^ and Ca^2+^ transport in Fpn. Fe^2+^ or Co^2+^ transport is mediated by both S1 and S2, and binding of Ca^2+^, which renders Asp39 unavailable, does not significantly affect Fe^2+^ or Co^2+^ transport because part of S1, His43, and the entire of S2 remain available to Fe^2+^ or Co^2+^ transport. On the other hand, Ca^2+^ transport is mediated by a single site and in the presence of Fe^2+^ or Co^2+^, Ca^2+^ binding site is impaired due to the loss of Asp39 and hence significantly reduced Ca^2+^ binding and transport (Figure 4—figure supplement 3a–c). Indeed, Asp39Ala single mutation significantly reduces Ca^2+^ transport (Figure 3a) but has a modest effect on Co^2+^ transport (Figure 3—figure supplement 3a–b). Further studies are needed to dissect the intertwined transport pathways of Fe^2+^ and Ca^2+^ transport in Fpn.

## Discussion

In summary, we show that HsFpn has a well-defined single Ca^2+^ binding site, and that HsFpn transports Ca^2+^. We also show that Ca^2+^ transport by HsFpn is not coupled to another ion and is diminished in the presence of Fe^2+^, however, Fe^2+^ transport is not sensitive to the presence of Ca^2+^.

Our report of Ca^2+^ transport by HsFpn contradicts the conclusion from a previous study (Deshpande et al., 2018); however, the two studies also have common grounds. Deshpande et al. identified a Ca^2+^ binding site in the structure of BbFpn (*Bdellovibrio bacteriovorus*), a bacterial homolog of human Fpn (Figure 2—figure supplement 3a), and demonstrated Ca^2+^ binding to mouse Fpn, which is in agreement with our demonstration of a Ca^2+^ binding site in HsFpn. Deshpande et al. conclude that Ca^2+^ may serve as a bound “cofactor” required for Fe^2+^ transport (Deshpande et al., 2018), and our results imply that Fpn could transport Fe^2+^ while bound to a Ca^2+^. With the structure of Ca^2+^-bound HsFpn, we noticed that residues forming the Ca^2+^ binding site are conserved between BbFpn and HsFpn, except for Asn100 in HsFpn, which is Phe85 in BbFpn. The persistence of a Ca^2+^ binding site from bacteria to mammals suggests a conserved functional role of Ca^2+^, and future studies aimed at understanding the biological relevance of Ca^2+^ transport through Fpn will lead to understanding of regulations in iron homeostasis.

Ca^2+^ binding and transport in HsFpn imposes constraint to the conceptualization of the Fe^2+^/2H^+^ antiport mechanism in the context of S1 and S2 of Fe^2+^ binding sites. In the absence of a bound Ca^2+^, export of one Fe^2+^ could be mediated through sequential occupation of S1 and S2 and structural changes that expose the binding sites to the extracellular side; and import of two H^+^ could be mediated by protonation of His43 at S1 and His507 at S2 followed by a structural change that exposes the two residues to the intracellular side. When Ca^2+^ is bound, Asp39 is not available to mediate Fe^2+^ export so that transport would likely be mediated by S2 only or by coalescing of His43 and S2 around a single Fe^2+^. Since we do not observe significant changes in Fe^2+^ transport in the presence and absence of Ca^2+^, it is likely that S1, or residue Asp39, has a more modest effect on Fe^2+^ transport, while S2 has a more prominent role. Results from our initial mutational studies on S1 and S2 are consistent with this hypothesis. Further studies are required to establish a mechanism of transport.

Our discovery of Ca^2+^ transport in Fpn demonstrates a novel Ca^2+^ entry pathway in cells expressing Fpn, and our study establishes Fpn as a transporter capable of operating with two different transport mechanisms. Although we are not able to estimate the amount of Ca^2+^ uptake through HsFpn under physiological conditions to provide an interpretation for its cellular function, we speculate that Ca^2+^ entry through HsFpn is an important component contributing to iron homeostasis.

## Materials and methods

**Key resources table:** 

Reagent type (species) or resource	Designation	Source or reference	Identifiers	Additional information
Chemical compound, druC	n-Dodecyl-β-D-Maltopyranoside	Anatrace	Cat#D310	
Chemical compound, drug	Lauryl maltose neopentyl glycol	Anatrace	Cat#NG310	
Chemical compound, drug	1-palmitoyl-2-oleoyl-glycero-3-phosphocholine (POPC)	Avanti Polar Lipids	Cat#850457 C	
Chemical compound, drug	1-palmitoyl-2-oleoyl-sn-glycero-3-phosphoethanolamine (POPE)	Avanti Polar Lipids	Cat#850457 C	
Chemical compound, drug	1-palmitoyl-2-oleoyl-sn-glycero-3-phospho-(1'-rac-glycerol) (POPG)	Avanti Polar Lipids	Cat#840457 C	
Chemical compound, drug	Calcein	Invitrogen	Cat#C481	
Chemical compound, drug	Fluo-4, Pentapotassium Salt, cell impermeant	Invitrogen	Cat#F14200	
Chemical compound, drug	Valinomycin	Sigma	Cat#V0627	
Peptide, recombinant protein	Hepcidin-25 (human)	Sigma	Cat#SML1118	
Commercial assay, kit	TALON Metal Affinity Resin	TaKaRa	Cat#635504	
Commercial assay, kit	SRT-10C SEC-300	Sepax Technologies	Cat#239300–10030	
Commercial assay, kit	Q Sepharose Fast Flow	GE Healthcare	Cat#17051010	
Commercial assay, kit	Biobeads SM2	Bio-Rad	Cat#1528920	
Commercial assay, kit	PD-10 Desalting Column	GE Healthcare	Cat#17085101	
Commercial assay, kit	400 nm NanoSizer Extruder	T&T Scientific Corporation	Cat#TT-004–0010	
Commercial assay, kit	KOD Hot Start DNA polymerase	Novagen	Cat#71086–3	
Commercial assay, kit	Fluo-4, AM, cell permeant	Invitrogen	Cat#F14201	
Commercial assay, kit	pHrodo Red AM	Invitrogen	Cat#P35372	
Recombinant DNA reagent	pGP-CMV-jGCaMP7s	Addgene	Plasmid #104463	RRID:Addgene_104463
Software, algorithm	MotionCorr2	Zheng et al., 2017	https://msg.ucsf.edu/em/software/motioncor2.html	
Software, algorithm	Gctf	Zhang, 2016	https://www.mrc-lmb.cam.ac.uk/kzhang/Gctf/	RRID:SCR_016500
Software, algorithm	Relion 3.0	Kimanius et al., 2016	https://www3.mrc-lmb.cam.ac.uk/relion	RRID:SCR_016274
Software, algorithm	CryoSPARC	Punjani et al., 2017	https://cryosparc.com/	RRID:SCR_016501
Software, algorithm	ChimeraX	Pettersen et al., 2021	https://www.cgl.ucsf.edu/chimerax/	RRID:SCR_015872
Software, algorithm	COOT	Emsley et al., 2010	https://www2.mrc-lmb.cam.ac.uk/personal/pemsley/coot/	RRID:SCR_014222
Software, algorithm	PHENIX	Adams et al., 2010	http://www.phenix-online.org/	RRID:SCR_014224
Software, algorithm	EMringer	Barad et al., 2015	http://fraserlab.com/2015/02/18/EMringer/	
Other	Grids: R1.2/1.3 Cu 300 mesh	Quantifoil	Cat#Q325CR1.3	Cryo-EM grid

### Cloning, expression, and purification of human Fpn (HsFpn)

Codon-optimized cDNA of HsFpn (UniProt ID: Q9NP59) was cloned into a pFastBac dual vector. A Tobacco Etch Virus (TEV) protease site and an octa-histidine (8×His) tag was appended to the C-terminus of the protein. HsFpn was expressed in Sf9 (*Spodoptera frugiperda*) cells using the Bac-to-Bac method (Invitrogen). Purification of HsFpn follows the same protocol reported for *Tarsius syrichta* Fpn (TsFpn) (Pan et al., 2020). Purified HsFpn was collected from size-exclusion chromatography (SEC) in the FPLC buffer containing 20 mM 4-(2-Hydroxyethyl)piperazine-1-ethanesulfonic acid (HEPES, pH7.5), 150 mM NaCl, and 1 mM (w/v) n-dodecyl-β-D-maltoside (DDM, Anatrace). Mutations to HsFpn were generated using the QuikChange method (Stratagene) and verified by sequencing. Mutants were expressed and purified following the same protocol for the WT.

### Preparation of HsFpn-11F9 complex in nanodisc

Membrane scaffold protein (MSP) 1D1 was expressed and purified following an established protocol (Martens et al., 2016). For lipid preparation, 1-palmitoyl-2-oleoyl-glycero-3-phosphocholine (POPC, Avanti Polar Lipids), 1-palmitoyl-2-oleoyl-sn-glycero-3-phosphoethanolamine (POPE, Avanti Polar Lipids) and 1-palmitoyl-2-oleoyl-sn-glycero-3-phospho-(1'-rac-glycerol) (POPG, Avanti Polar Lipids) were mixed at a molar ratio of 3:1:1, dried under Argon and resuspended with 14 mM DDM (Autzen et al., 2018). For nanodisc reconstitution, HsFpn, MSP1D1 and lipid mixture were mixed at a molar ratio of 1:2.5:50 and incubated on ice for 1 hr. Detergents were removed by the sequential addition of 60 mg/mL Biobeads SM2 (Bio-Rad) for three times over a 3-hr period. The sample was then incubated with Biobeads overnight at 4 °C. After removal of Biobeads, 11F9 was added to the sample at a molar ratio of 1.1:1 to HsFpn. The complex was incubated on ice for 30 min before being loaded onto a SEC column equilibrated with the FPLC buffer without detergent. The purified nanodisc sample was concentrated to 10 mg/ml and incubated with 2 mM CaCl_2_ for 30 min on ice before cryo-EM grid preparation.

### Cryo-EM sample preparation and data collection

The cryo-EM grids were prepared using Thermo Fisher Vitrobot Mark IV. The Quantifoil R1.2/1.3 Cu grids were glow-discharged with air for 15 s at 10 mA using Plasma Cleaner (PELCO EasiGlow). Aliquots of 3.5 µl nanodisc sample were applied to the glow-discharged grids. After blotted with filter paper (Ted Pella, Inc) for 4.0 s, the grids were plunged into liquid ethane cooled with liquid nitrogen. A total of 4498 micrograph stacks were collected on a Titan Krios at 300 kV equipped with a K3 direct electron detector (Gatan) and a Quantum energy filter (Gatan) at a nominal magnification of 81,000×and defocus values from –2.5 µm to –0.8 µm. Each stack was exposed in the super-resolution mode with an exposing time of 0.0875 s per frame for a total of 40 frames per stack. The total dose was about 50 e^-^/Å^2^ for each stack. The stacks were motion corrected with MotionCor2 (Zheng et al., 2017) and binned 2-fold, resulting in a pixel size of 1.10 Å/pixel. In the meantime, dose weighting was performed (Grant and Grigorieff, 2015). The defocus values were estimated with Gctf (Zhang, 2016).

### Cryo-EM data processing

A total of 2,184,301 particles were automatically picked based on a reference map of TsFpn-11F9 (EMD-21460) low-pass filtered to 20 Å in RELION 3.1 (Scheres, 2015, Scheres, 2012; Kimanius et al., 2016; Zivanov et al., 2018). Particles were extracted and imported to CryoSparc (Punjani et al., 2017) for 2D classification. A total of 1,329,782 particles were selected from good classes in 2D classification, which display recognizable structural features. Four 3D references were generated by *ab initio* reconstruction with limited particles from the best 2D classes. Multiple rounds of heterogeneous refinement were performed with particles selected from the 2D classification and four initial reference models until no more than 5% of input particles were classified into bad classes. A final of 437,959 particles after heterogeneous refinement were subjected to non-uniform (NU) refinement with an adaptive solvent mask. After handedness correction, local refinement and CTF refinement were performed with a soft mask around the Fpn and the Fv region of the Fab. Resolutions were estimated with the gold-standard Fourier shell correlation 0.143 criterion (Rosenthal and Henderson, 2003). Local resolution of the maps was estimated in CryoSparc (Punjani et al., 2017).

### Model building and refinement

The structure of apo HsFpn (from PDB ID 6W4S) and 11F9 Fab (from PDB ID 6VYH) were individually docked into density maps in Chimera (Pettersen et al., 2004). The docked model was manually adjusted with added ligands in COOT (Emsley et al., 2010). Structure refinements were carried out by PHENIX in real space with secondary structure and geometry restraints (Adams et al., 2010). The EMRinger Score was calculated as described (Barad et al., 2015). All structure figures were prepared in Pymol and ChimeraX (Pettersen et al., 2021).

### Isothermal titration calorimetry

The WT and mutant HsFpn proteins were purified as described above and concentrated to 50–75 μM (3–4.5 mg/mL) in the FPLC buffer. The buffer was degassed, and all the protein samples were centrifuged at 18,000×g for 20 min to remove aggregates. The injectant of 2 mM CaCl_2_ or 5 mM CoCl_2_ was prepared in the same FPLC buffer. For competition binding, either 2 mM CoCl_2_ or CaCl_2_ was added to protein samples prior to ligand titration. The ITC measurements were performed in Auto-iTC200 (MicroCal) at 25 °C. A total of 25 injections were administered (1.01 μL for injections 1 and 2.02 μL for injections 2–25) with a 150 s interval between injections. Background-subtracted data were fitted with binding models in the Origin 8 software package (MicroCal) to extract *K_D_*, ΔH, and entropy change (ΔS).

### Expression of HsFpn in HEK cells

The cDNAs of WT and mutant HsFpn were subcloned into a modified pEG BacMam vector with a C-terminal Strep-tag. The resulting plasmids with Fpn or the empty plasmid were transfected into HEK 293 F cells on six-well plates with 293fectin transfection reagent (Invitrogen/Thermo Fisher) per the manufacturer’s protocol. After incubation at 37 °C with 8% CO_2_ for 2 days, cells were harvested and solubilized in the lysis buffer (20 mM HEPES, pH 7.5, 150 mM NaCl, 10% glycerol) plus 1% LMNG and Protease Inhibitor Cocktail (Roche) for 1 hr at 4 °C. Insoluble fractions were pelleted by centrifugation and supernatants were run in SDS-PAGE. Proteins were visualized by western blotting with mouse anti-Strep (Invitrogen/Thermo Fisher) and IRDye-800CW anti-mouse IgG (Licor). Images were taken on an Odyssey infrared scanner (Licor).

### Ca^2+^ uptake and H^+^ transport assays in HEK cells

The pEG BacMam plasmids with HsFpn or the empty plasmid were transfected into HEK 293 F cells on black wall 96-well microplates coated with poly-D-lysine (Invitrogen/Thermo Fisher). After 2 days, cells were washed in the live cell imaging solution (LCIS) containing 20 mM HEPES (pH 7.4), 140 mM NaCl, 2.5 mM KCl, 1.0 mM MgCl_2_, and 5 mM D-glucose. The loading of Fluo-4 (Invitrogen/Thermo Fisher, AM, cell-permeant) for Ca^2+^ uptake assays or pHrodo Red (Invitrogen/Thermo Fisher, AM) for H^+^ transport assays was performed following manufacturer’s protocols. After the loading finished, free dyes were washed away, and cells in each well were maintained in 90 µL LCIS. Both the Ca^2+^ uptake and H^+^ transport assays were performed in the FlexStation 3 Multi-Mode Microplate Reader (Molecular Devices) at 37 °C. Fluorescence changes were recorded at an excitation and emission wavelength of 485 nm and 538 nm for Ca^2+^ uptake assays, and 544 nm and 590 nm for H^+^ transport assays with 5 s intervals. Transport was triggered by the addition of 10 µL ligand stock solution (CaCl_2_ or CoCl_2_) to achieve the desired concentration of extracellular Ca^2+^ or Co^2+^. The H^+^ transport was assayed with 500 µM Co^2+^ or Ca^2+^. To test the effect of extracellular pH on Ca^2+^ uptake, the extracellular buffer was changed to pre-warmed LCIS with adjusted pH soon before the addition of 500 µM Ca^2+^. All the mutants were assayed with 500 µM Ca^2+^. For Ca^2+^ uptake assays, the initial rate is defined by slope of a linear fit to the first 25 s of data points. For H^+^ transport assays, relative fluorescence changes at the equilibrium stage were averaged to represent intracellular pH changes.

### Ca^2+^ uptake in the presence of Fe^2+^ or Co^2+^ in HEK cells

The WT and mutant HsFpn were expressed in HEK 293 F cells as described above except that the plasmid for mammalian expression of jGCaMP7s (pGP-CMV-jGCaMP7s) was co-transfected. The pGP-CMV-jGCaMP7s was a gift from Douglas Kim & GENIE Project (Addgene plasmid # 104463; http://n2t.net/addgene:104463; RRID:Addgene_104463) (Dana et al., 2019). GCaMP is a green fluorescence protein (GFP)-based Ca^2+^ indicator that contains a fused calmodulin (CaM) domain. Ca^2+^ binding to CaM triggers conformational changes that results in increased GFP fluorescence. Ca^2+^ uptake in the presence of Fe^2+^ or Co^2+^ were performed in FlexStation 3 as described above except that the excitation and emission wavelength were set at 485 nm and 513 nm. Fe^2+^ (NH_4_Fe(SO_4_)_2_) or Co^2+^(CoCl_2_) ions were loaded into cells by incubation for ~10 min at 37 °C, during which time fluorescence readings were recorded. For Fe^2+^ loading, 1 mM sodium ascorbate was used to protect Fe^2+^ ions from oxidation. To start the export of Fe^2+^ or Co^2+^, the extracellular buffer was exchanged to pre-warmed LCIS ~30 s before the addition of Ca^2+^. LCIS with adjusted pH was used when testing the effect of extracellular pH. The initial rate is defined as the slope of a linear fit to the first 25 s of transport data.

### Reconstitution of HsFpn into liposomes

POPE and POPG lipid (Avanti Polar Lipids) were mixed at a 3:1 molar ratio, dried under Argon, and vacuumed overnight to remove chloroform. The dried lipid was resuspended in the reconstitution buffer (20 mM HEPES, pH 7.5, 100 mM KCl) to a final concentration of 10 mg/mL. After hydration for 2 hr, the liposome sample was sonicated to transparency and incubated with 40 mM n-decyl-β-D-maltoside (DM, Anatrace) for 2 hr at room temperature under gentle agitation. Then HsFpn protein was added at a 1:100 (w/w, protein:lipid) ratio. For the empty control, the same volume of blank buffer was added. Detergent was removed by dialysis at 4 °C against the reconstitution buffer. Dialysis buffer was changed every day for 4 days. The proteoliposome or empty liposome sample was aliquoted and frozen with liquid nitrogen, and was stored at –80 °C for future use.

### Ca^2+^ and Fe^2+^/Co^2+^ influx in proteoliposomes

Proteoliposomes with HsFpn or empty liposomes were thawed and mixed with 100 μM Fluo-4 (Invitrogen/Thermo Fisher, cell impermeant) for Ca^2+^ influx assays, or with 250 μM calcein (Invitrogen/Thermo Fisher) for Fe^2+^/Co^2+^ influx assays. The dye was incorporated during three cycles of freeze-thaw. Liposomes were extruded to homogeneity with a 400 nm filter (NanoSizer Extruder, T&T Scientific Corporation). Removal of free dyes outside liposomes and exchange of outside buffer was achieved by passing samples through a desalting column (PD-10, GE Healthcare) equilibrated with buffer required for a desired transport condition. Liposome samples were transferred to a quartz cuvette for fluorescence recording in a FluoroMax-4 spectrofluorometer (HORIBA). Fluorescence changes were recorded at an excitation and emission wavelength of 494 nm and 513 nm with 10 s intervals at 37 °C.

To monitor Ca^2+^ influx, transport was initiated by the addition of CaCl_2_ to the desired concentration. When testing the inhibition by hepcidin or 11F9 Fab (i)/(o), 20 µM human hepcidin (Sigma) or purified 11F9 Fab (Pan et al., 2020) was added prior to freeze-thaw cycles, and the transport was assayed with 500 µM CaCl_2_. For the Fab (o) condition, 20 µM Fab was added to samples collected from desalting column. When testing the effect of different pHs, an outside buffer has the same components as the inside buffer but was adjusted to a desired pH. When testing the effect of different Na^+^, K^+^, or Cl^-^, the inside and outside of liposomes have symmetrical buffers with 100 mM NaCl, KCl, or K-Gluconate. For measurements with –120 mV membrane potential shown in Figure 1—figure supplement 4a, the outside buffer has 20 mM HEPES (pH 7.5), 1 mM KCl, and 99 mM NaCl while the inside buffer has 20 mM HEPES (pH 7.5) and 100 mM KCl. 40 nM valinomycin was added to clamp membrane potential at ~–120 mV. When testing the effects of different membrane potentials, valinomycin was incubated with liposome samples for 5 min prior to the addition of CaCl_2_. For the –120 mV group, the outside buffer contained 20 mM HEPES (pH 7.5), 1 mM KCl, and 99 mM NaCl while the inside buffer has 20 mM HEPES (pH 7.5) and 100 mM KCl. For the 0 mV group, the outside buffer was the same as the inside buffer with 20 mM HEPES (pH 7.5) and 100 mM KCl. For the +120 mV group, the inside buffer has 20 mM HEPES (pH 7.5), 1 mM KCl, and 99 mM NaCl while the outside buffer contained 20 mM HEPES (pH 7.5) and 100 mM KCl.

For Fe^2+^/Co^2+^ influx assays, symmetrical buffer (20 mM HEPES, pH 7.5, 100 mM KCl) was used and transport was initiated by dilution (10×) of 30 µL of liposome sample into 270 µL of outside buffer containing 50 µM of NH_4_Fe(SO_4_)_2_ or CoCl_2_. In the case of Fe^2+^ influx, 1 mM sodium ascorbate was added. To load liposomes with Ca^2+^, 1.25 mM or 0.5 mM CaCl_2_ was added prior to freeze-thaw cycles. Buffer with 1.25 mM or 0.5 mM CaCl_2_ was used during the desalting step. To create a 10-fold Ca^2+^ gradient opposite to the Fe^2+^ or Co^2+^gradient, samples were diluted into the outside buffer without Ca^2+^.

## Data Availability

The cryo-EM density map of nanodisc-encircled HsFpn-11F9 in the presence Ca2+ has been deposited in the Electron Microscopy Data Bank (https://www.ebi.ac.uk/pdbe/emdb/) under accession code EMD-27497. The corresponding atomic coordinate file has been deposited in the Protein Data Bank (http://www.rcsb.org) under ID code 8DL6. Uncropped gel and blot images are available as source files. The following datasets were generated: ShenJ WilbonAS PanY ZhouM 2023Cryo-EM structure of human ferroportin/slc40 bound to Ca2+ in nanodiscRCSB Protein Data Bank8DL6 PanY ShenJ WilbonAS ZhouM 2023Cryo-EM structure of human ferroportin/slc40 bound to Ca2+ in nanodiscArrayExpressEMD-27497

## Additional files

MDAR checklist
