# Supplementary figures and images for: Mechanism of Ca2+ transport by ferroportin
Source: eLife. 2023 Jan 17;12:e82947. doi: 10.7554/eLife.82947 (PMC9883014; doi:10.7554/eLife.82947)

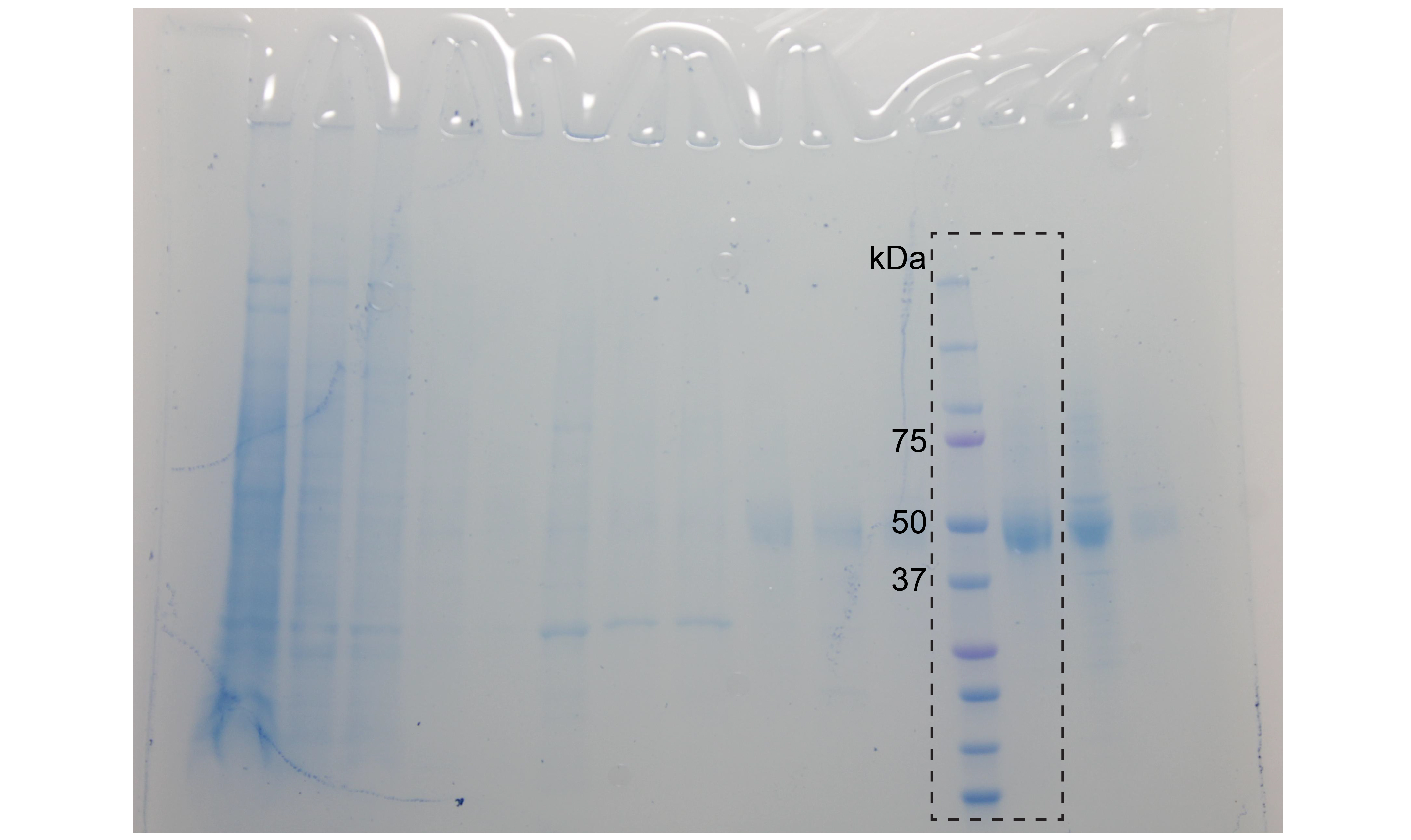

Supplement: Figure 1—figure supplement 1—source data 1. — Black dashed rectangle indicates the region of interest. [file elife-82947-fig1-figsupp1-data1.zip › Figure 1ΓÇöfigure supplement 1ΓÇösource data 1.jpg]

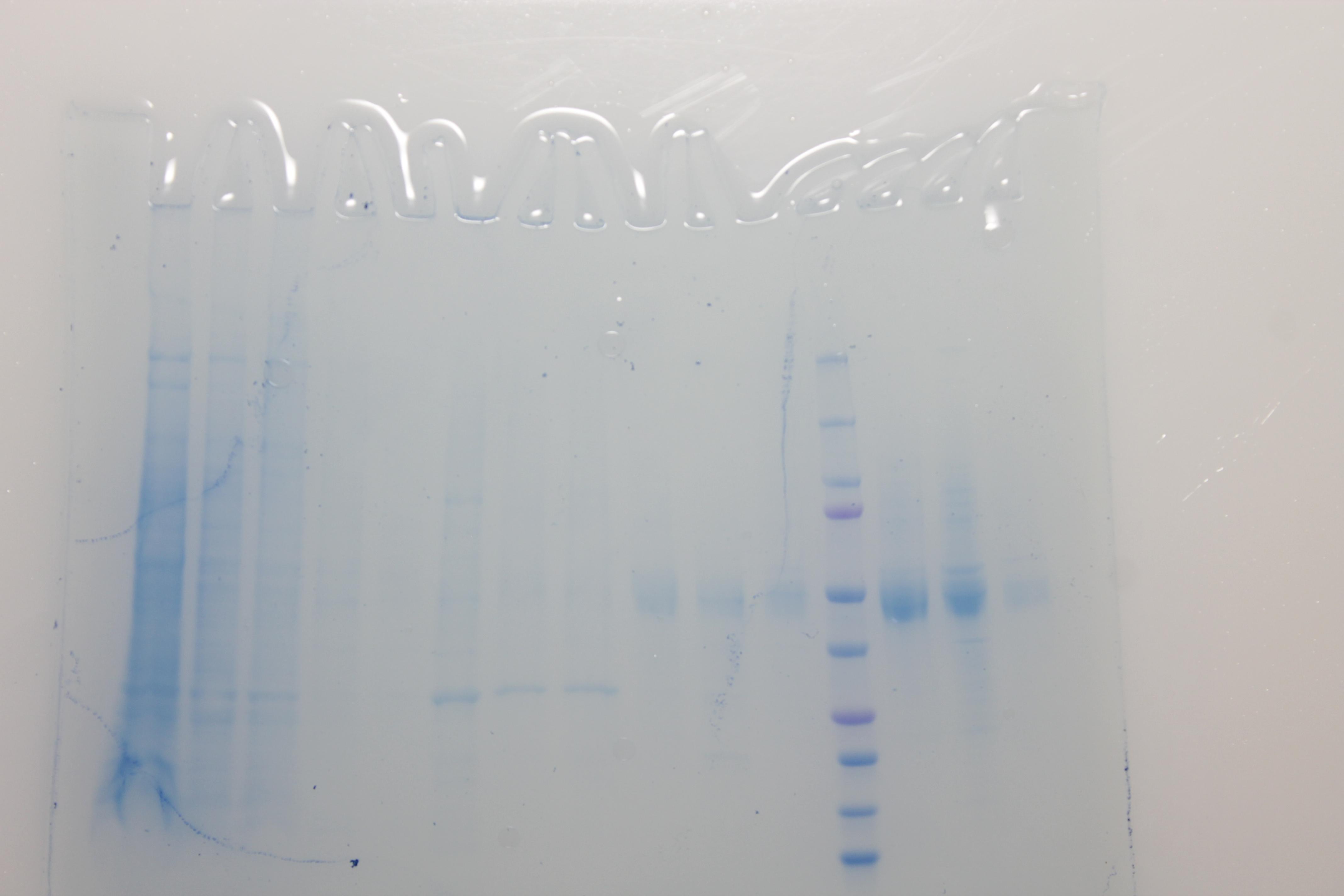

Supplement: Figure 1—figure supplement 1—source data 1. — Black dashed rectangle indicates the region of interest. [file elife-82947-fig1-figsupp1-data1.zip › Fpn-SDS-PAGE.JPG]

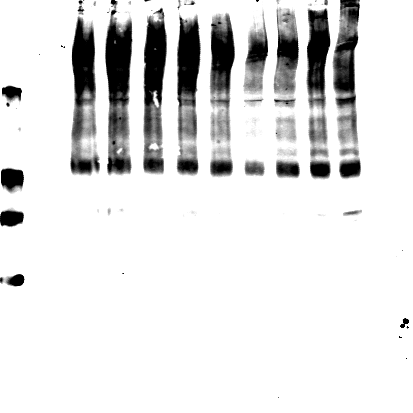

Supplement: Figure 3—figure supplement 1—source data 1. — Black dashed rectangle indicates the region of interest. [file elife-82947-fig3-figsupp1-data1.zip › Fpn-western-raw.tif]

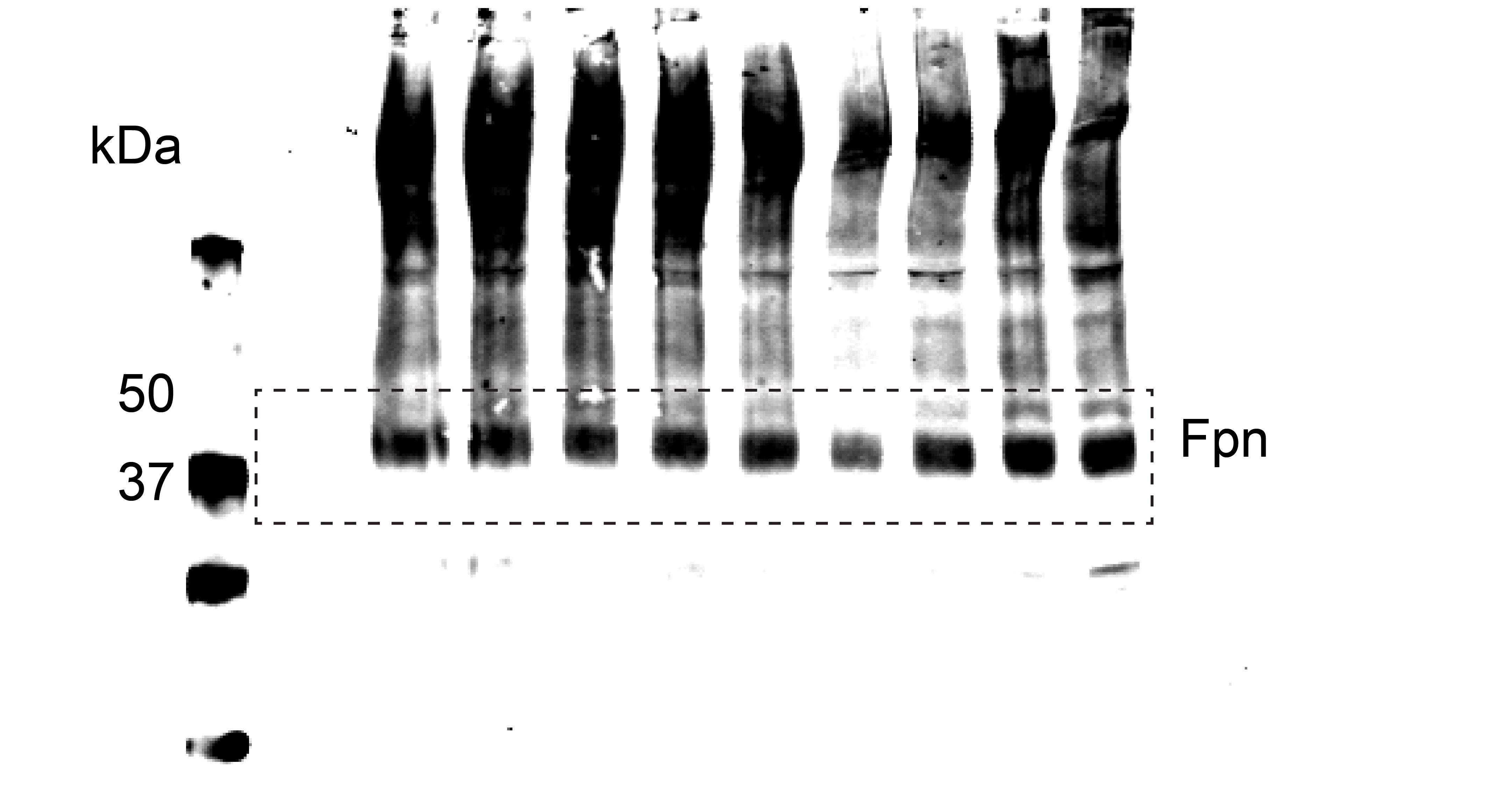

Supplement: Figure 3—figure supplement 1—source data 1. — Black dashed rectangle indicates the region of interest. [file elife-82947-fig3-figsupp1-data1.zip › Figure 3ΓÇöfigure supplement 1ΓÇösource data 1.jpg]
