# Supplementary material for: Mechanism of Ca2+ transport by ferroportin
Source: eLife. 2023 Jan 17;12:e82947. doi: 10.7554/eLife.82947 (PMC9883014; doi:10.7554/eLife.82947)
Supplement: Peer review history

# Editor report: Editor's evaluation

**DOI:** 10.7554/eLife.82947.sa0

**Authors:** David Drew

**Affiliations:** https://ror.org/05f0yaq80 Stockholm University Sweden

This study makes an important contribution to the function of the iron transporter Ferroportin (Fpn). By using a combination of proteoliposome assays, mutagenesis and structural studies by cryo EM, the authors are able to demonstrate that the H^+^-driven transporter for Fe2+-efflux is also capable of passive Ca^2+^ influx. The evidence supporting the conclusions is convincing, but the physiological relevance of Ca^2+^ entry by Fpn is yet to be established. The work will be of broad interest to cell biologists and biochemists.

# Decision letter

**DOI:** 10.7554/eLife.82947.sa1

**Affiliations:** https://ror.org/05f0yaq80 Stockholm University Sweden; https://ror.org/05f0yaq80 Stockholm University Sweden; https://ror.org/043mz5j54 University of California, San Francisco United States

Our editorial process produces two outputs: (i) public reviews designed to be posted alongside the preprint for the benefit of readers; (ii) feedback on the manuscript for the authors, including requests for revisions, shown below. We also include an acceptance summary that explains what the editors found interesting or important about the work.

**Decision letter after peer review:**

Thank you for submitting your article "Mechanism of Ca^2+^ transport by ferroportin" for consideration by *eLife*. Your article has been reviewed by 3 peer reviewers, including David Drew as the Reviewing Editor and Reviewer #1, and the evaluation has been overseen by Kenton Swartz as the Senior Editor. The following individual involved in review of your submission has agreed to reveal their identity: Aashish Manglik (Reviewer #3).

The reviewers have discussed their reviews with one another, and the Reviewing Editor has drafted this to help you prepare a revised submission.

Major concerns:

You have been able to robustly demonstrate Ca^2+^ binding. What is unclear is if Ca^2+^ is actually transported to a significant level? Is the Fpn protein just capable of one turnover in proteoliposomes? We highly recommend kinetics using 45CaCl2 (eg. PNAS E5354-E5362) to calculate actual Ca^2+^ turnover (kcat).

*Reviewer #1 (Recommendations for the authors):*

The authors have been able to robustly demonstrate Ca^2+^ binding. What is unclear to me is how much Ca^2+^ is actually transported? Is the Fpn protein just capable of one turnover in proteoliposomes? The "cleanest" experiment would be to measure Ca^2+^ uptake using 45CaCl2 (eg. PNAS E5354-E5362) so one can calculate Vmax and kcat. From my understanding, metalloproteins do not necessarily use the optimum metal for catalysis, but the metal available in the appropriate concentration in where they function. I wonder if the Ca^2+^ site is just not selected against, because its not needed too, i.e., Ca^2+^ doesn't compete for Fe2+ uptake and the actual "turnover" for Ca^2+^ is very low?

*Reviewer #2 (Recommendations for the authors):*

The data reported here appear to be of high quality and are convincing; the experimental design is excellent and the necessary controls are appropriately employed. A couple of issues need clarification in the text. FPN is clearly an iron efflux pump and these studies make clear that Fpn can also import Ca^2+^, although it does not appear to function as an Fe2+-Ca^2+^ antiporter. What is less clear is whether Fpn will transport calcium bi-directionally. There are technical issues that would make this a bit hard to study, but if the authors think the answer is clear from their data, they should be explicit in explaining this. A further question that needs explaining is why bind and transport calcium? Cells have a high capacity for calcium flux independent of Fpn. Is there a physiological importance to this activity?

# Reply: Author response

**DOI:** 10.7554/eLife.82947.sa2

Major concerns:

You have been able to robustly demonstrate Ca^2+^ binding. What is unclear is if Ca^2+^ is actually transported to a significant level? Is the Fpn protein just capable of one turnover in proteoliposomes? We highly recommend kinetics using 45CaCl2 (eg. PNAS E5354-E5362) to calculate actual Ca^2+^ turnover (kcat).

Uptake of Ca^2+^ is measured by changes of Fluo-4 fluorescence. The fluorescence intensity increases progressively as we increase the concentrations of the Ca^2+^ and reaches up to five-fold higher than the initial value. As shown in Author response 1, a five-fold increase of fluorescence intensity requires >20 µM of Ca^2+^. If we assume that a lipid headgroup has an area of ~60 Å2 and that liposomes have an average diameter of ~400 nm, we arrive at a rough estimate of >100 turnovers for each Fpn under 500 µM external Ca^2+^ as shown in Figure 1—figure supplement 4a.

**Author response image 1.** Titration of Ca^2+^ to Fluo-4 used in liposome transport assays. (a) Emission spectra of 100 µM Fluo-4 in the inside buffer at 37 °C titrated with increasing [Ca^2+^] (from blue to red). (b) Correlation of F/F0 to [Ca^2+^]. The blue line is the linear fit to data in the region of [Ca^2+^] within 2 µM.

Reviewer #1 (Recommendations for the authors):

The authors have been able to robustly demonstrate Ca^2+^ binding. What is unclear to me is how much Ca^2+^ is actually transported? Is the Fpn protein just capable of one turnover in proteoliposomes? The "cleanest" experiment would be to measure Ca^2+^ uptake using 45CaCl2 (eg. PNAS E5354-E5362) so one can calculate Vmax and kcat. From my understanding, metalloproteins do not necessarily use the optimum metal for catalysis, but the metal available in the appropriate concentration in where they function. I wonder if the Ca^2+^ site is just not selected against, because its not needed too, i.e., Ca^2+^ doesn't compete for Fe2+ uptake and the actual "turnover" for Ca^2+^ is very low?

See response to Major concerns.

Reviewer #2 (Recommendations for the authors):

The data reported here appear to be of high quality and are convincing; the experimental design is excellent and the necessary controls are appropriately employed. A couple of issues need clarification in the text. FPN is clearly an iron efflux pump and these studies make clear that Fpn can also import Ca^2+^, although it does not appear to function as an Fe2+-Ca^2+^ antiporter. What is less clear is whether Fpn will transport calcium bi-directionally. There are technical issues that would make this a bit hard to study, but if the authors think the answer is clear from their data, they should be explicit in explaining this. A further question that needs explaining is why bind and transport calcium? Cells have a high capacity for calcium flux independent of Fpn. Is there a physiological importance to this activity?

We added an explanation that the Fpn in liposomes is randomly oriented and made a schematic drawing (Figure 1—figure supplement 1c) to illustrate this point. We also measured Ca^2+^ transport when the Fab was added only to the external side, and we found that the rate of transport is higher than the rate when the Fab was added to both sides and lower than the rate when no Fab was added (Figure 1—figure supplement 1f–g). We take this result as further indication that Fpn is reconstituted in both orientations.

We are also intrigued by the calcium transport activity of Fpn, and more study is required to address the questions on biology Ca^2+^ uptake by Fpn. We speculate that loss of cellular Fe^2+^ will lead to Ca^2+^ influx through Fpn, which may trigger further cellular responses.

Although our study arrived at a different conclusion from that of Deshpande et al. 2018 with regard to Ca^2+^ transport, both studies share common grounds on the calcium binding site and transport of Fe^2+^ in the presence of Ca^2+^. We made a comment on this in Discussion.
